# *CFTR*-Related Metabolic Syndrome: Genetic Variants Increasing Pancreatitis Risk in the Pediatric Puerto Rican Population

**DOI:** 10.3390/children10020280

**Published:** 2023-01-31

**Authors:** Jesús M. Meléndez-Montañez, Wilfredo De Jesús-Rojas

**Affiliations:** Department of Pediatrics and Basic Science, Ponce Health Sciences University, Ponce, PR 00716, USA

**Keywords:** cystic fibrosis, *CFTR*-related metabolic syndrome, chronic/recurrent pancreatitis, *CFTR* gene, *CFTR*-related disease, pancreatic insufficiency

## Abstract

*CFTR*-related metabolic syndrome (CRMS) is a novel diagnosis due to widespread use of and advances in the newborn screening (NBS) process for cystic fibrosis (CF) in the United States of America, allowing for the diagnosis of asymptomatic children with CF. Before 2015, a large Puerto Rican pediatric population was not screened for CF in the NBS test. Studies have shown that patients presenting with idiopathic recurrent or chronic pancreatitis have an increased frequency of cystic fibrosis transmembrane conductance regulator (*CFTR*) gene mutations. We present a retrospective chart review of 12 pediatric cases (*n* = 12) that were presented to an outpatient community clinic with clinical manifestations associated with CF. The pancreatic insufficiency prevalence (PIP) score was calculated on *CFTR* mutations. The mutations considered for the calculation of the PIP score were: F508del (c.1521_1523del), V201M (c.601G > A), I507del (c.1519_1521del), and L1335P (c.4004T > C). V201M mutation was classified as mild in both PIP scores, and a correlation with pancreatitis was noted. Clinical manifestations vary in cases with the V201M variant (c.601G > A). One case was diagnosed with *CFTR*-related disorder (CRD) and recurrent pancreatitis. It is important to consider CRMS or CRD as a differential diagnosis in the pediatric population of Puerto Rico due to the implications and increased risk of pancreatitis and other CF-related complications.

## 1. Introduction

Advances in genetics, the implementation of newborn screening (NBS), and the availability of sweat tests allow for early diagnosis of children with cystic fibrosis (CF) and *CFTR*-related metabolic syndrome (CRMS) in the United States of America. When a CF screening test does not meet the established criteria as per CF guidelines [1], other entities, such as CRMS and *CFTR*-related disorder (CRD), should be a diagnostic consideration as part of the differential diagnosis in a patient with clinical symptoms. It is suggested that the term CRMS be used to refer to newborns with hypertrypsinogenemia on the NBS who have sweat chloride readings <60 mmol/L and up to two *CFTR* variants, at least one of which is not characterized as a CF-disease-causing variant, and who do not match the criteria set out by the CF Foundation for the diagnosis of CF [2]. CRD is a nonclassical CF induced by disease-causing variants of different severity, resulting in either single or multiorgan involvement, depending on each organ's susceptibility to malfunctioning of the cystic fibrosis transmembrane conductance regulator (CFTR) protein [3]. In Puerto Rico, it is estimated that the incidence of CF is one in every 17,000 births. Since 2015, it has been mandatory by law to include an immunoreactive trypsinogen (IRT) test to screen for CF as part of the NBS at all hospitals around the island. However, a large portion of the Puerto Rican pediatric population was not tested for CF in the NBS in Puerto Rico before 2015. As a result, there is a hypothetical likelihood of having undiagnosed CF in the adolescent and adult Puerto Rican population. In addition, until 2021, there was just one laboratory on the island that could perform the analysis of sweat samples, currently the gold standard for CF diagnostic confirmation. 

Patients presenting with recurrent-acute or chronic pancreatitis have an increased frequency of mutations in the *CFTR* gene [4]. A small percentage of this group can fulfill the criteria for CF diagnosis, and the remaining individuals who do not fulfill the criteria could be diagnosed with CRD in the presence of *CFTR* variants [5]. The pancreatic insufficiency prevalence (PIP) score is a practical approach to understanding how patients who carry *CFTR* variants with mild phenotypic effects have a greater risk of developing pancreatitis than those associated with moderate-to-severe phenotypes. This *CFTR* genotype–phenotype correlation found in pancreatitis is unique compared to other organ manifestations related to the complex monogenic nature of the CF disease [6]. As a result of the related long-term implications, clinical suspicion of CRMS or CRD should be explored in pediatric patients who suddenly develop idiopathic recurrent or chronic pancreatitis. The purpose of this research is to provide a description of the disease-causing variations of the *CFTR* gene that are now elevating the risk of pancreatitis in the Puerto Rican pediatric population.

## 2. Materials and Methods

We present a retrospective chart review of 12 pediatric cases (*n* = 12) referred to an outpatient community clinic specialized in the evaluation of rare pediatric pulmonary disorders in Puerto Rico. Each pediatric patient was previously or recently diagnosed, as per CF Foundation guidelines [1], with having either CF, CRMS, or CRD, or classified as a CF carrier. Failure to thrive (FTT) was defined by a body mass index (BMI) below the 3rd percentile for age. The diagnosis classification process included a review of sweat chloride assays, as well as genetic testing for *CFTR* variants. Genetic testing results were analyzed regarding the base change, protein/intron position, zygosity, and variant classification. An electronic chart review of the enzymatic measurements of lipase, amylase, and pancreatic stool elastase levels was assessed when available. Based on the reference values, patients were classified as either exocrine pancreatic insufficient (EPI) (<200 mcg/g stool elastase) or exocrine pancreatic sufficient (EPS) (>200 mcg/g stool elastase). The pancreatic insufficiency prevalence (PIP) score was calculated and compared to those in the CFTR2 database for the same mutations. *CFTR* mutations found were explored in the Clinical and Functional Translation of CFTR (CFTR2) database from patients from the United States and around the world [7]. An analysis of the *CFTR* variant name, exocrine pancreatic insufficiency (EPI) patients, exocrine pancreatic sufficiency (EPS) patients, and the total number of patients with each variant in the database was completed. The *CFTR* variants in the cohort with a PIP score of ≤0.25 were classified as mild. The PIP score was also calculated for CFTR2 patients and compared with those found in our retrospective cohort of pediatric patients from Puerto Rico. The Institutional Review Board of the Ponce Health Sciences University in Ponce, Puerto Rico approved data collection and analysis for the protection of human subjects. 

## 3. Results

From a total of 12 pediatric patients (*n* = 12), seven were males (58%) and five were females (42%) with a median age of 7.55 years and Puerto Rican ethnicity. The most frequently reported findings were FTT, recurrent pancreatitis, and abnormal IRT. The complete data list for *CFTR* base change, protein/intron position, zygosity, and mutation classification is presented in Table A1. In some cases, data on sweat tests, lipase levels, amylase levels, and pancreatic stool elastase were unavailable. A total of six EPS cases were reported, three EPI, and in three, data were unavailable (Table 1).

The mutations F508del (c.1521_1523del), V201M (c.601G > A), I507del (c.1519 1521del), and L1335P (c.4004T > C), which were present in two or more cases, were considered for the calculation of the PIP score. F508del obtained a PIP score of 0.25 in our cohort and 0.88 in the CFTR2 database, V201M PIP scores of 0.00 and 0.23, I507del PIP scores of 1.00 and 0.89, and finally, L1335P PIP scores of 0.50 and 0.37. V201M mutation was classified as mild in both PIP scores of 0.00 and 0.23. The CFTR2 database had a total of 53,480 EPI patients for F508del, 3 EPI patients for V201M, 529 EPI patients for I507del, and 11 EPI patients for L1335P. Our cohort had one EPI patient for F508del, zero EPI patients for V201M, two EPI patients for I507del, and finally, one EPI patient for L1335P, which are presented in Table 2 and Table 3.

## 4. Discussion

CF is a clinical diagnosis due to the broad spectrum and severity of symptoms and organs affected amongst individuals [8,9,10,11]. CF can also be defined genetically by the presence of biallelic CF-disease causing variants in the *CFTR* gene. Physiologically, it is a disorder of chloride and bicarbonate transport through epithelial membranes resulting from the absence or inappropriate functioning of the CFTR protein [9]. CRMS or CRD should be a diagnostic consideration in the appropriate clinical setting when a clinical entity associated with CFTR dysfunction does not meet the criteria of CF guidelines [1,11]. Gastrointestinal manifestations in CRMS and CRD are important to be addressed by clinicians due to the potential implications for children's nutrition, growth, and overall development.

Recurrent-acute or chronic pancreatitis illustrates an accurate phenotype of CFTR dysfunction [12]. CF-disease-causing variants, such as F508del being the most frequent and severe, generally have less than 2% CFTR protein function, leading to EPI in homozygote patients. However, CF cases with mild genotypes that have more than 5% of normal CFTR function are considered EPS [11]. A published systematic study of 277 EPS with CF participants with or without pancreatitis demonstrated that the risk of developing pancreatitis was linked to the severity of the *CFTR* genotype [13]. After PIP score calculation, patients with mild mutations had a greater risk of developing pancreatitis than those with moderate or severe *CFTR* genotypes [14]. In the literature, the V201M missense variant of *CFTR* and other missense mutations of *CFTR* have not yet been identified for their role in gene expression and function [15]. In our study, the V201M mutation was considered mild; similarly, the CFTR2 database classifies the V201M mutation as mild. In contrast, F508del was classified as mild in our cohort, going against the CFTR2 database. This discrepancy could be attributed to the small sample size of our retrospective study, which limited our observations. Another reason for the discrepancy found in our cohort could be non-CFTR factors that affect and modify, both genetically and environmentally, the phenotype and clinical progression of the pancreatic function of this Puerto Rican cohort with F508del versus those found in the CFTR2 database [16,17]. 

In our study cohort, the I507del and L1335P variations were classed as moderate, as they are in the CFTR2 database. Cases 7 and 9 in our study cohort presented with the V201M variant (c.601G > A) but clinical manifestations, physical findings, and diagnoses varied between the two cases. Case 7 was diagnosed with CRMS, and clinical manifestations and physical findings were remarkable for the history of recurrent pancreatitis. Currently, case 7 is EPS, and the diagnosis of pancreatitis increases her risk of developing EPI in the future [18]. The exact pathogenesis of the development of EPI is still unknown and under investigation, but the leading cause is thought to be obstructive tubulopathy due to CFTR channel dysfunction in the pancreatic duct [19]. Case 9 was diagnosed as a CF carrier and asymptomatic. It is important for asymptomatic CF carriers with specific mutations to be monitored long-term as studies show that CF carriers exhibit slight CFTR dysfunction and are at risk of CRD and, therefore, chronic pancreatitis in heterozygous carriers [20]. Case 5 was remarkable for recurrent pancreatitis, the EPS status was maintained, and the F508del was considered mild under the PIP score in our cohort compared to the CFTR2 database. This observation is consistent with the complex nature of CF, particularly in children, in whom all CF manifestations may or may not be present at the beginning. Over time, we can expect that the damage to the pancreatic acinar tissue will cause EPI in most cases [21]. As recommended by CF Foundation guidelines, in pediatric cases, close monitoring and serial clinical evaluations will help us understand and detect EPI and recurrent pancreatitis in populations with less common *CFTR* genetic mutations, such as in Puerto Rico [22]. To address the increased risk of pancreatitis and CF among Puerto Rican children, a collaborative and multidisciplinary strategy involving a pediatrician, gastroenterologist, pulmonologist, geneticist, and dietician with experience in CF, CRD, and CRMS will be required. Table 4 provides a suggested guide for the multidisciplinary approach and clinical investigations necessary to evaluate patients with CF, CRD, and CRMS in Puerto Rico.

It is essential to continue monitoring and consider CRMS or CRD as a differential diagnosis in the pediatric population of Puerto Rico with complaints such as failure to thrive, abdominal pain, and pancreatitis. A considerable portion of the Puerto Rican pediatric population was not screened for CF prior to 2015 since IRT levels were not yet included in the NBS. At the same time, access to sweat tests on the island was limited, with only one laboratory previously available. As a result, adolescents may be at risk of complications of CFTR dysfunction, such as pancreatitis, in Puerto Rico. Our team has recently established a second laboratory for sweat test collection and analysis, which has been operating since 2021. This facility adheres to the guidelines established by the CF Foundation, makes use of cutting-edge equipment, and is fully accredited by the Clinical Laboratory Improvement Amendments (CLIA). Table 5 summarizes the challenges of studying and treating CF and CF-related pancreatitis in the Puerto Rican population.

All these efforts will help us to increase awareness about the disease, which may help to identify and recruit more patients for research in Puerto Rico. In the pediatric population, the risk of malignancy is low. However, as the age of survival continues to rise, there is an increased risk of malignancy in the gastrointestinal and biliary tracts for patients with *CFTR* variants associated with an increased risk of pancreatitis [23]. Insight into the molecular mechanisms relating to *CFTR* variants and functions is much needed to understand and develop targeted drug treatments for underrepresented populations in research. Currently, there is no specific therapy for CF-related pancreatitis, but new and highly effective CFTR modulator therapies aiming to modulate and potentiate the CFTR function may have implications for specific mutations affecting the gastrointestinal component of patients with CF-related pancreatitis and EPI [24,25]. 

Similarly, challenges with CF identification are lower in North American and European countries compared to Latin American nations due to a lack of official patient registries, universal access to newborn screening, up-to-date diagnostic technologies, and interdisciplinary and specialized clinical centers [26]. Table 6 summarizes the published studies evaluating *CFTR* variants in Latin America. Specific screening panels customized to their population's genetic profile were researched in Ecuador, demonstrating the need to study Puerto Rico's genetic profile to understand our unique *CFTR* variability [27]. However, doing so in Latin America has been difficult in recent decades due to the resources required for molecular testing and the poor detection rates of known *CFTR* variants [28,29]. Prior epidemiologic studies aimed at identifying CF patients and genetic variations in different parts of the nation were necessary for understanding CF in Latin America [28,30]. The Health State Department manages the Puerto Rican CF incidence, and no epidemiological research has been conducted on the CF Puerto Rican population. Only one study documented the frequency of *CFTR* variants in Puerto Rico and the Dominic Republic, showing an increased variability of the *CFTR* variants, with a high potential to be missed by the newborn screening [22]. In the same study, the most frequent *CFTR* variant for Puerto Rico was F508del, congruent with our observations. A cross-sectional investigation in Uruguay found that the incidence of CF was significantly lower than the state authorities claimed [31]. Additional studies are needed to determine the incidence of CF in Puerto Rico as the NBS was not mandated by legislation in Puerto Rican hospitals before 2015 and probably missed uncommon *CFTR* genetic variants in our population, especially in adults.

As a result of the heterogeneity of CF, a registry of *CFTR* variants is required, as demonstrated by a study in Colombia [32], to tailor specific screening tests for common variants such as F508del and other rarer and understudied *CFTR* variants to the mixture of European, Amerindian, and African ancestry in many Latin American countries such as Puerto Rico. According to research, Mexico has one of the most diversified *CFTR* spectra in the world [33]. This trend of genetic variability in Latin American countries was demonstrated in other studies, where the F508del was present but at lower rates than in European countries [34]. The presence of rare mutations with the calculation of the PIP score may have health implications in these countries, as demonstrated in our Puerto Rican cohort with pancreatitis. A better understanding of the ancestry of *CFTR* variants in Latin America may help to clarify the complexity and segregation of rare *CFTR* variants in our communities. 

## 5. Conclusions

Our study cohort of 12 Puerto Rican pediatric patients demonstrated the presence of *CFTR* variants causing recurrent pancreatitis and EPI in Puerto Rico. We described the association between V201M *CFTR* mutation and CRMS diagnosis with a greater risk of developing chronic pancreatitis in Puerto Ricans. High clinical suspicion of Puerto Rican pediatric and adult patients presenting with recurrent pancreatitis is needed between healthcare providers to consider CRMS or CRD in the differential diagnosis. CF screening is even more critical considering that, before 2015, NBS tests were not mandatory for CF in Puerto Rico, and a large portion of the pediatric population was not screened for an elevation of IRT nor the most common mutations of the *CFTR* gene. For this reason, many patients may continue to go undiagnosed with chronic pancreatitis in adulthood. Early diagnosis allows for a better prognosis and quality of life for CF, CRMS, and CRD patients, along with CF carriers. Additional basic, clinical, and translational research in underrepresented minority groups, such as patients living in Latin America, is needed to develop specific treatments for CF-related pancreatitis. A better understanding of the CF genotype–phenotype relationship is necessary for Hispanic populations such as Puerto Ricans, especially in children. Teamwork among pediatric pulmonologists and general pediatricians in Puerto Rico is vital to raise awareness about CF and to enable us to fully understand the *CFTR* spectrum of disease. 

## Figures and Tables

**Table 1:** Laboratory measurements of pancreatic function of 12 Puerto Rican pediatric patients with *CFTR* variants.

Case #	Diagnosis	Lipase (U/L)	Amylase (U/L)	Sweat Test (R/L)	Pancreatic Stool Elastase (mcg/g)	Exocrine Pancreatic Function ^3^
1	CF carrier	62	55	**	**	**
2	CF carrier	**	**	20/20	>200	EPS
3	CF ^1^	10	34	101/97	<10	EPI
4	CRMS ^2^	65	117	36/35	349	EPS
5	CF ^1^	64	64	65/63	>500	EPS
6	CF ^1^	**	**	37/39	>500	EPS
7	CRMS ^2^	310	181	33/30	>500	EPS
8	CF carrier	**	**	10/10	483	EPS
9	CF carrier	**	**	11/12	**	**
10	CF carrier	**	**	**	**	**
11	CRMS ^2^	82	63	31/34	66	EPI
12	CF^1^	**	**	136/131	<50	EPI

** Data unavailable.^1^ CF: cystic fibrosis.^2^ CRMS: *CFTR*-related metabolic syndrome. Pancreatic stool elastase (normal > 200 mcg/g) (severe < 100 mcg/g). ^3^ Cases were classified as either exocrine pancreatic insufficient (EPI) or exocrine pancreatic sufficient (EPS) based on the reference values of pancreatic stool elastase (normal > 200 mcg/g or severe < 100 mcg/g).

**Table 2:** Pancreatic insufficiency prevalence (PIP) score calculated on *CFTR* variants present two or more times in our Puerto Rican pediatric cohort.

*CFTR* Variant	Variant Name	PIP Score	EPS Cases	EPI Cases
c.1521_1523del	F508del	0.25 *	3	1
c.601G > A	V201M	0.00 *	2	0
c.1519_1521del	I507del	1.00	0	2
c.4004T > C	L1335P	0.50	1	1

* Based on the PIP score, variants that obtained a score of ≤0.25 were classified as mild variants.

**Table 3:** Pancreatic insufficiency prevalence (PIP) score calculated on *CFTR* mutations present two or more times in our Puerto Rican pediatric cohort using the CFTR2 database to compare.

*CFTR* Variant	Variant Name	PIP Score	EPS Cases	EPI Cases
c.1521_1523del	F508del	0.88	7293	53480
c.601G > A	V201M	0.23 *	10	3
c.1519_1521del	I507del	0.89	65	529
c.4004T > C	L1335P	0.61	18	11

* Based on the PIP score, variants that obtained a score of ≤ 0.25 were classified as mild variants.

**Table 4:** Pediatric multidisciplinary approach and clinical investigations for CF, CRD, and CRMS in Puerto Rico.

Healthcare Provider	Investigations	Evaluation
Pediatrician	Monitor weight, stool patterns, follow up NBS * results	BMI percentile, referral to sub-specialties
Geneticist	Familial pedigree	*CFTR* gene testing
Pulmonologist	Access for pulmonary symptoms	Spirometry, chest X-ray, sweat test
Gastroenterologist	Screening for pancreatitis and EPI, constipation/diarrhea	Abdominal X-ray, lipase, and amylase levels, fecal fat and pancreatic stool elastase levels
Dietitian	Access fiber intake and promote adequate hydration	Calculate daily caloric intake

* NBS: newborn screening, EPI: exocrine pancreatic insufficiency.

**Table 5:** Challenges to studying and treating CF and CF-related pancreatitis in Puerto Rico.

Challenge	Action
Lack of an accredited CF center in Puerto Rico	Establish an IRB-approved patient registry for all CF patients on the island examined by pulmonologists and gastroenterologists, including pediatric and adults; develop a multidisciplinary clinic in accordance with the standards of the CF Foundation
Decreased awareness about CF and CF-related pancreatitis among the general community and medical providers	Improve healthcare professionals’ involvement in CF-related webinars and medical congresses and encourage the Puerto Rico Department of Health to support ongoing medical education about rare diseases prevalent on the island, such as CF; promote the implementation of CF screening red flags in schools; encourage the referral of patients with FTT and CF-related symptoms for *CFTR* testing
Lack of subspecialties on the island with research training and medical expertise in CF	Promote interest in CF and related research among medical students, along with CF-related careers as a subspecialty; partner with a CF center in the United States to provide educational opportunities for medical students and healthcare providers practicing on the island; empower current providers with research education to enable them to study CF and other rare diseases
Chronic illnesses, such as asthma, are common in Puerto Rico, and may co-exist with and act as a confounder for CF	Increase the referral rate among general practitioners for patients with asthma and other CF-related symptoms such as FTT, recurrent pancreatitis, chronic constipation or diarrhea, nasal polyps, or a positive family history of CF
Limited funding opportunities to study rare diseases such as CF	Encourage local and national research organizations to include rare diseases such as CF as part of Puerto Rico's research funding priorities

**Table 6:** Summary of published studies evaluating *CFTR* variants in Latin America.

Study Referenced	Study Design	Number of Subjects	Country	Genetic Variants	Major Finding
Ortiz et al., 2017 [27]	Prospective	48 patients	Ecuador	p.F508del, p.G85E, p.G330E, p.A455E, p.G970S, W1098X, R1162X, N1303K	The following variants should be included in the screening panel for the Ecuadorian population with cystic fibrosis: p.F508del, p.G85E, p.G330E, p.A455E, p.G970S, W1098X, R1162X, and N1303K.
Orozco et al., 2000 [29]	Prospective	97 families	Mexico	W1098C, P750L, 846delT, 4160insGGGG 297-1G-->A p.F508del	The detection rate of CF variants in Mexico remains lower than in other groups with a low frequency of the p.F508del variant, mostly from southern Europe.
Silva et al., 2016 [26]	Short communication	Not applicable	Mexico Argentina Brazil Chile Cuba	Not applicable	Although significant progress has been made, this communication has identified several areas where significant work remains to be done, including the development and maintenance of CF patient registries; universal access to neonatal screening and up-to-date diagnostic tools; more uniform input from multidisciplinary and specialized teams; improvements in provisions for adults with CF; and improved access to treatment.
Perez et al., 2007 [28]	Meta-analysis	2177 patients	Argentina Brazil Chile Colombia Costa Rica Cuba Ecuador Mexico Uruguay Venezuela	p.F508del, p.G542X, p.R1162X, p.N1303K, p.W1282X	It is challenging to find CF variants through molecular testing in most Latin American countries. Determining the molecular genetic epidemiology of CF in different countries is essential to schedule more precise molecular diagnostics for patients and make studies more cost-effective, especially when money is a limitation.
Cardoso et al., 2004 [31]	Cross-sectional	500 patients	Uruguay	p.F508del	The actual rate of incidence in the Uruguayan population is substantially lower (6.9 cases per 100,000 people) than prior estimates of 25 occurrences per 100,000 people.
Keyeux et al., 2003 [32]	Prospective	92 patients	Colombia	p.F508del, c.1811 + 1.6kbA > G, p.G542X, p.S549R, p.W1282X, p.R1162X	Findings indicate that a detailed examination of the *CFTR* variants is required in each region of Latin America to build priority-screening processes tailored to each country and region.
Chavez-Saldaña et al., 2010 [33]	Cross-sectional	133 patients	Mexico	p.R334W, p.A455E, c.3120 + 1G > A, c.3272-26A > G c.711 + 1G > T, p.Q552X, p.W1282X, c.IVS8-5T, p.R1162X, p.R347P, p.D1152H, p.T1036N	According to this study, Mexico has one of the world's most diverse *CFTR* mutation spectra.
Faucz et al., 2010 [30]	Meta-analysis	Unknown	Brazil	p.F508del, p.G542X, p.N1303K, p.G551D, p.R553X	The prevalence of European, African, and Amerindian ethnic groupings in modern Brazilian CF patients is shown by the *CFTR* variant spectrum. In this paper, researchers report an analysis of *CFTR* allelic heterogeneity and analyze the origins of its genetic makeup to provide insights for CF population screening and genetic counseling in Brazil.
Restrepo et al., 2000 [34]	Prospective	192 alleles	Mexico Colombia Venezuela	p.F508del, G542X, N1303K, 3849 + 10kb C > T	Although the frequency of DeltaF508 described in these Latin American countries is lower than in Caucasian populations, including in Spain, G542X and 3849 + 10kb C > T variants' prevalence are found to resemble those observed in Spain.
Venegas et al., 2003 [34]	Prospective	24 patients	Costa Rica	p.F508del, G542X	This study indicates significant disparities in Costa Rican CF genotypes when compared to other North American and European groups, as well as American Hispanics, raising crucial questions concerning isolated founder effects and population screening efforts in Costa Rica.
Zeiger et al., 2020 [22]	Prospective	82 patients	Dominic Republic Puerto Rico	p.F508del, p.Arg1066Cys, p.Arg334Trp, p.I507del, p.Ala559Thr	In the first description of *CFTR* variants in Caribbean countries, Dominic Republic, and Puerto Rico, 10% of Puerto Ricans have unknown *CFTR* variants.

## Data Availability

All data analyzed in this study are included in this published article.

## Appendix A

**Table A1:** Clinical manifestations and *CFTR* variants of 12 Puerto Rican patients.

Case #	Diagnosis	Clinical Manifestations	Base Change	Protein/Intron Position	Zygosity	Classification
1	CF carrier	12-year-old female with recurrent pulmonary infections and abdominal pain	c.1230-34TG(12)T(5)	Intron 9	Heterozygous	Pathogenic
2	CF carrier	3-year-old male with FTT and asthma	c.4004T > C c.1210-34TG(11)T(5)	p.Leu1335Pro Intron 9	Heterozygous Heterozygous	Pathogenic Pathogenic (low penetrance)
3	CF	17-year-old male with FTT, osteoporosis, bronchiectasis, and clubbing	c.1519_1521del	p.Ile507del	Heterozygous	Pathogenic
			c.1521_1523del	p.Phe508del	Heterozygous	Pathogenic
4	CRMS	30-month-old male with FTT and recurrent pneumonia	c.1521_1523del	p.Phe508del	Heterozygous	Pathogenic
5	CF	12-year-old with FTT and chronic pancreatitis	c.1521_1523del	p.Phe508del	Heterozygous	Pathogenic
6	CF	19-months-old with FTT and chronic pancreatitis	c.1521_1523del	p.Phe508del	Heterozygous	Pathogenic
			c.601G > A	p.Val201Met	Heterozygous	Pathogenic
7	CRMS	9-years-old female with FTT and recurrent pancreatitis	c.601G > A	p.Val201Met	Heterozygous	Pathogenic
8	CF carrier	33-months-old male with chronic cough	c.1210-34TG(11)T(5)	Intron 9	Heterozygous	Pathogenic (low penetrance)
9	CF carrier	15-months-old female with abnormal IRT	c.1000C > T	p.Arg334Trp	Heterozygous	Pathogenic
10	CF carrier	20-year-old male asymptomatic	c.601G > A	p.Val201Met	Heterozygous	Pathogenic
11	CRMS	9-year-old male with FTT and chronic bronchitis	c.4004T > C	p.Leu1335Pro	Heterozygous	Pathogenic
			c.1210-34TG(11)T(5)	Intron 9	Heterozygous	Pathogenic (low penetrance)
12	CF	7-month-old male with FTT, steatorrhea, anemia, and pneumothorax	c.1519_1521del	p.Ile507del	Heterozygous	Pathogenic
			c.3196C > T	p.Arg1066Cys	Heterozygous	Pathogenic

CF: cystic fibrosis, CRMS: *CFTR*-related metabolic syndrome, FTT: failure to thrive, IRT: immunoreactive trypsinogen.
